# Birdlime

**Published:** 1901-11-09

**Authors:** 


### THE INSTITUTIONAL WORKSHOP.

### BIRDLIME.

SOME NOTES ON THEIR APPLICATION FOR CATCHING RATS, MICE, AND OTHER CARRIERS OF INFECTIOUS GERMS. BY S. VERNON KAY.

THE interest which a short letter of mine, which appeared in THE HOSPITAL of October 12th, has aroused emboldens me to write this short article in continuance of the subject in the hope that it may be of service to those interested, and perhaps induce such scientists as are engaged in the prevention of the spread of infection by means of flies, vermin, etc., to experiment in this direction, and give their attention to a simple means of catching these pests which has not received the consideration it deserves.

Birdlime has been known from the earliest times. In the olden days in England it used to be made from the bark of the holly, which was stripped from the trees at a certain time of the year, boiled, pounded, washed, and fermented until a kind of grease resulted, which floated to the surface. This was skimmed off and kept in jars until required for use, the chief customers for the article being gamekeepers and birdcatchers, who found it useful in their callings. Instances are known of even such varied creatures as hawks, weasels, moles, stoats, and even cats being caught by it.

There are many varieties of birdlime, nearly every country having its own special kind, and they vary considerably both as regards colour, composition, and effectiveness. It must be borne in mind, however, that it does not follow that the birdlime which is most effective in one country is necessarily the best in another. In fact, it is rather the other way. Climate has everything to do with it. A birdlime to be used in England, or similar temperate climates, requires its greatest adhesive powers to be active between, say, 60° and 80°. In Spain, for example, one is required which shall be most effective between 70° and 90°. In North and South Africa the temperature is very much higher in the season, and a birdlime to be of any service must successfully resist a temperature of 100° to 110°.

The Japanese birdlime is well known. This is a light, khaki-coloured substance of great adhesiveness and good non-running properties. It is very suitable for hot climates, but little use for cold ones. Its great drawback is its liability to oxidise, when it becomes black in colour and useless. Dak birdlime is very similar in appearance to Jap, and is most suitable for colonial use. It possesses the advantage, however, of being less liable to oxidation than Jap. It also gives a more even smear, and is less variable in consistency than the latter, which is seldom obtained twice alike and varies from the consistency of putty to a watery mush.

In Spain the natives use an article they call "Ajonje.' It is manufactured from the "Ajojera," a species of thistle plant, Euphorbia, that grows on the hills. In this case the birdlime is made from the seeds, which are collected in July. This "Ajojera" birdlime is, however, of very poor quality. It is mouse-colour in appearance, and is generally in jelly-like lumps. Its tenacity is very poor, and both Dak and Jap are far superior to it.

There is a Hungarian variety, also made from a seed. This produces a dirty-yellow-looking material, of very offensive smell. It is little used; in fact it is hardly good enough for any purpose. And so it is everywhere. Even the Moors have a birdlime of their own; sometimes one sees it for sale in the market-places; but this is similar in composition to that of the Spaniards, and is weak and of little utility.

For use in England the thick green "Cheshire" birdlime is found the most effective, and this is the kind used generally. It is a good bird, rat, and fly catcher, and answers the demands of the climate. In hot weather, however, it becomes somewhat thin, and is therefore not suitable for export, as at temperatures when "Dak" or "Jap" would be doing well "Cheshire" would be too soft to be effective.

The disappearance of the poisonous flypaper and its replacement by the harmless sticky flypaper has led to the adoption of various birdlimes, compositions and mixtures, and flygums specially prepared for this purpose can now be obtained. Birdlime has been used for flypapers for many years, but it is only of late that the manufacture of these has been improved, and an article turned out neat and clean enough for general use.

Flies are everywhere. Indeed, "the rank fly" corruptious insect is universally abominated. When we remember that one fly is capable of laying 2,000,000 eggs it is not to be wondered at that there are so many. If every fly lived its life we should soon be crushed off the earth. However, my purpose in writing this article is not to comment on flies, but rather to explain how they may be caught. From the researches of Yersin, Wyman, and other scientists it seems now proved beyond all reasonable doubt flies are great carriers of plague infection. Yersin found in his laboratory dead flies whose bodies were crowded with bacilli. The bacilli are also found on the bodies of rats and mice found dead during the epidemic. Now where does birdlime come in?

First, with regard to rats. If it is desired to make a colony desert their burrows, it is only necessary to smear a little round the entrances. If it is desired to catch them, the best way is to dress plenty of straws and spread them thickly upon the ground around the burrows. Among the straws throw some attractive bait, malt sprinkled with oil of carraway is a good draw. When the spot is visited next morning the straws will be found gathered up in little bundles, and in the centre of each will be a rat, alive or dead, according to the extent of its entanglement. Do not be tempted to place your foot upon one of these rats if it still struggles, but kill it with a stick. The same *modus operandi* is employed in catching mice. If the birdlime is to be used indoors, take a big piece of stiff brown paper instead of straw, and put the sticky stuff in the centre. In England I would recommend "Cheshire," abroad "Jap" or "Dak." And caution—when you go to see the bag don't take your dog, or the last stage may be worse than the first. A birdlimed dog is not in a condition to be of use to anybody. By the way, nearly all birdlimes are readily soluble in mineral naphtha or paraffin, and should any get on the clothes it may readily be removed by either of these.

No article on birdlime would be complete without directions for use. Naturally it is awkward stuff to handle for those not accustomed to it. First of all then it is necessary to understand that birdlime will not adhere to anything wet; hence the hands and scissors should always be kept in that state. The knife should be kept dry. Usually birdlime is supplied in tins, but in small quantities; for flycatching, etc., it is best to get it in collapsible tubes. In this latter case, of course, it is just squeezed out. When bought in tins, however, it is better to warm gently on the hob before proceeding to work. This thins it. Then remove the lid and dig the point of a dry knife into it. The birdlime sticks to the blade and can then be drawn out in ropes. Cut off smartly near the tin with the wet scissors, and then smear with the knife on the paper or string, or whatever it is required to cover.

And now for flies. I have experimented personally with all kind of sticky flycatchers in all sorts of countries and climates, and I find that the first desideratum is a first-class gum. Birdlime itself is too thick for general use, and too easily oxidises when exposed in a thin layer to direct sunlight. A user will not be far wrong if he uses flypapers made by any well-known English maker, as the gums used on these are the outcome no doubt of considerable experience. They are so cheap, and are packed so neatly, that it is rarely necessary to make ones own, though in foreign places where it is not possible to buy them a tin of birdlime or flygum can be kept and smeared on papers as required. Two or three hundred flies is a small catch for a good paper. For catching quickly, however, nothing appears so effective as a suspensory flycatcher. Flies always congregate around anything hanging, and rest on it continually. Notice the chandelier at home. If a sticky string or wire be used, the settling becomes a permanency, and the flies rapidly decrease. With this object several flycatchers have been put on the market constructed on this principle; but the one I have tested and found to be the most effective is a spiral wire one. This has been put on the market under the name of the "Vampire," and at the present time I have letters from all parts abroad from medical men and others the former strongly recommending them for hospital use. This catcher as made up consists of a closely wound wire spiral covered with a suitable gum and hermetically sealed in a short tube. By pulling out one end the spiral is elongated, and when suspended forms a most effective flycatcher.

It may interest your readers to know that some hundreds of experiments were carried out before gums suitable to the various climates were worked out. In addition experiments have been carried on personally in France, Spain, and North Africa. Samples have also been tested and reported on in Australia, America, Canada, South America, and many other places abroad. I have seen one of these catchers with as many as 500 flies on it, and the average catch is 200 to 300.

They are being tested at present on the West Coast of Africa by one of the malarial expeditions for mosquito catching, and seems very probable that they can be used for this purpose. Even if the gum as used at present is not sufficiently attractive for the mosquito, it very possibly can be made so by the addition of a small quantity of blood or other attraction. I have tried making the gum luminous so as to show in the darkness; thinking this may attract, but up to the present I have no data as to the success of the experiment. Mosquitoes, thank goodness, do not trouble us here.

A Spaniard once informed me that a small cup of vinegar put by the bedside at night is sufficieut to draw the mosquitoes. He said that he had often tried it with effect, and on some occasions had made quite a good catch this way. I tried this one night, but I regret to say without effect. There was only one mosquito in the room, and apparently he did not care for vinegar. Still the statement is there, and perhaps some readers abroad may try the experiment in the season and obtain better results. A Moor, too, once informed me in confidence that a small piece of dried camel's dung burnt in a room will prevent the mosquitoes entering. This I have not tried.

I have given these disjointed facts for what they are worth. Perhaps this article may strike the eye of someone who is interested, and he may follow it up. I have not attempted to go into the medical aspect of the subject, which is not in my line. I look at it from the point of view of the chemist, endeavouring to find a satisfactory and cheap method of reducing these infection-carrying pests. If the information here given is of service my object is attained.

N.B.—Since the above article was written I have received a confirmation of the letter I append herewith. I received this some time ago, but seeing the subject was of importance I wrote asking Dr. Jeffreson to kindly confirm his letter with regard to mosquitoes. This he has done, assuring me that these flycatchers, filled with a special, non-running gum, are perfectly effective for mosquitoes, and so I now have no hesitation in making the fact public.

Tangier Hospital, Morocco, North Africa,
July 10th, 1901.

Dear Sir,—I cannot speak too highly of the flycatchers you sent me. I have used them both in the hospital and in private practice here, and they are a perfect blessing. There is no doubt in my mind, as a doctor, that it is a most useful and valuable hygienic invention and a preservative to health in tropical climates, attracting flies and mosquitoes at once on entering a house, which otherwise might be the means of spreading malarial fever or other tropical diseases. . . I shall most strongly advocate their use in the malarial fever season here, and use them in my house and any hospital I am connected with.

Yours faithfully,
(Dr.) J. Russell Jeffreson, F.R.G.S.

